# Effects of dietary minerals deficiency and supplementation on different parts of muscle minerals content in grazing Mongolian sheep

**DOI:** 10.3389/fvets.2024.1301852

**Published:** 2024-01-22

**Authors:** Lingbo Meng, Xiwei Jin, Zhi Qi, Lan Mi

**Affiliations:** State Key Laboratory of Reproductive Regulation and Breeding of Grassland Livestock, School of Life Sciences, Inner Mongolia University, Hohhot, China

**Keywords:** biceps femoris, longissimus dorsi, mineral, sheep, triceps brachii

## Abstract

**Objective:**

The objective of this study was to investigate the impact of dietary deficiency and supplementation of calcium, zinc, copper, cobalt, manganese or selenium on minerals content in the longissimus dorsi (LD), biceps femoris (BF) and triceps brachii (TB) of grazing Mongolian sheep.

**Methods:**

We randomly divided 98 sheep into 7 treatment groups and fed them specific diets for 60 days: a total mineral nutrition diet (LCG), a calcium deficiency diet (LCa), a zinc deficiency diet (LZn), a copper deficiency diet (LCu), a cobalt deficiency diet (LCo), a manganese deficiency diet (LMn) and a selenium deficiency diet (LSe). Then 7 sheep from each group were slaughtered and samples of LD, BF and TB were collected for mineral content analysis. The remaining sheep in each group were subsequently fed specific diets for an additional 41 days: a total mineral nutrition diet (SCG), a calcium supplementation diet (SCa), a zinc supplementation diet (SZn), a copper supplementation diet (SCu), a cobalt supplementation diet (SCo), a manganese supplementation diet (SMn) and a selenium supplementation diet (SSe). Afterward, all sheep were slaughtered, and muscle samples were collected and analyzed.

**Results:**

Significant findings emerged that LCa decreased sulfur (S) content in BF and increased Ca content in LD and BF, while SCa increased S and Ca content in BF and TB, respectively (*P* < 0.05). LZn decreased Zn, S, and potassium (K) content in LD and BF, while SZn increased Zn and S content in LD and BF, respectively (*P* < 0.05). LCu decreased Cu and iron (Fe) content in LD and TB, while SCu increased Fe content in TB (P < 0.05). LCo decreased phosphorus, S, K, Ca, Mn, Fe, Cu, and Zn content in LD (*P* < 0.05). LMn decreased Mn content and increased K content in TB, while SMn decreased K content in BF and TB (*P* < 0.05). LSe and SSe decreased and increased Se content in LD, BF, and TB, respectively (*P* < 0.05).

**Conclusion:**

Dietary mineral levels have varying effects on lamb meat minerals content. It is important to ensure an adequate intake of minerals in the diet to enhance the mineral nutrition of lamb meat.

## 1 Introduction

With the improvement of living standards for humans, the focus of demand for food has shifted from quantity to quality, which makes the high-quality food with local characteristics ever more popular (1). Inner Mongolia Autonomous Region's unique geographical advantages and conditions of grazing-based production have contributed to the Inner Mongolia grazing mutton, a characteristic local food. The grazing mutton is a kind of highly nutritious meat food because of its high protein, low cholesterol, low fat and rich minerals (2). Therefore, the composition of minerals composition in grazing mutton are also considered an important index to evaluate the quality.

Calcium (Ca), zinc (Zn), copper (Cu), cobalt (Co), manganese (Mn), and selenium (Se) are essential nutrients for various metabolic and physiological processes in the human body and animals (3). Nearly 99% of the Ca absorbed in animals is directed to the formation of bones and teeth, with the remaining 1% involved in a variety of physiological processes (4). Ca deficiency has the greatest impact on bone development and predisposes to osteoporosis (5). Zn is a component of DNA and RNA synthase as well as various digestive enzymes (6). At the same time, Zn also plays an important role in the human immune system, and insufficient Zn intake can cause immune dysfunction and decreased immunity (7). Cu is a critical component of tyrosinase and cytochrome C oxidase in animals, which are involved in physiological processes such as melanin metabolism and antioxidant defense (8). Reports suggest that inadequate Cu intake in infants may lead to impaired cardiovascular development and neurodevelopmental abnormalities (9). Co is an essential component of vitamin B12 in the body, and cobalamin (vitamin B12) regulates the production of red blood cells in the body, so Co deficiency can lead to anemia (10). Mn is related to a variety of enzymes in organisms that are involved in urea cycle and proteoglycan synthesis in cartilage (11). Therefore, Mn deficiency can also result in cartilage dysplasia for human (12). Se participates in a variety of life activities such as redox reaction, nervous system development and immune defense response through selenoprotein (13). Se deficiency affects a person's reproductive function and immune system, causing brain damage and cardiovascular-related diseases (14). As is well-known, these essential mineral nutrients for the human body cannot be synthesized internally and can only be obtained from diet (15). The abundant minerals in grazing mutton serve as a high-quality source of mineral nutrition that the human body requires (2).

In recent years, with overgrazing and grassland degradation, there has been a widespread deficiency of Ca, Zn, Cu, Co, Mn, and Se in grazing Mongolian sheep in the Inner Mongolia region (16–18). However, the effect of the levels of these minerals in the diet on the mineral composition of grazing Mongolian sheep meat remains unclear. Based on an understanding of existing research, there are various approaches that can be employed to investigate the response of grazing sheep meat to the levels of minerals in the diet. For example, these approaches may include the addition of inorganic mineral salts (19), organic mineral salts (20), and Nanominerals (21) to the diet. However, taking into account the actual conditions of grazing in the region, we have decided to use the addition of inorganic mineral salts to the diet as our research method. Therefore, this study focused on grazing Mongolian sheep as the research subjects, and the representative muscles of the sheep, inclusing the longissimus dorsi (LD), biceps femoris (BF), and triceps brachii (TB), were selected as the experimental material (22). We investigated the pattern of change in mineral nutrition in LD, BF, and TB by feeding deficiency and supplementation diets of Ca, Zn, Cu, Co, Mn or Se to 98 Mongolian sheep successively. In contrast to previous studies, our research focuses on the grazing sheep population for the first time and attempts to provide a comprehensive analysis of the effects of single mineral deficiencies and additions in the diet on the mineral content of different muscle tissues in grazing Mongolian sheep. This provides a reference for improving the quality of grazing mutton and meeting the human demand for mineral nutrition.

## 2 Materials and methods

All the animal-related procedures involved in this study were approved by the Animal Care and Use Committee of Inner Mongolia University (IMU-sheep-2020-040).

### 2.1 Animals and experimental design

The grazing Mongolian sheep used in this study were healthy 4-month-old sheep farmed under natural grazing conditions. They were purchased from Nao Muhan Gacha, Abaga Banner, Xilin Gol League, Inner Mongolia Autonomous region, China.

Ninety-eight Mongolian sheep were individually housed in the same 98 pens, respectively. The whole feeding period, with crushed oats as concentrate fodder and mix pasture (natural grassland of Xilin Gol League) as roughage, with free access to water. The minerals contained in the feed are detailed in Table S1. Ninety-eight sheep were adapted to the environment for ~28 days and fed with 200 g concentrate fodder and 400 g roughage on a daily basis for each sheep. Then, a 60-day mineral deficiency feeding period was started. These sheep were randomly divided into seven experimental groups: a control group of mineral deficiency feeding phase (LCG), a Ca deficiency group (LCa), a Zn deficiency group (LZn), a Cu deficiency group (LCu), a Co deficiency group (LCo), a Mn deficiency group (LMn), and a Se deficiency group (LSe). Each experimental group was supplemented with a mixed mineral salt according to the NRC standards (23), as detailed in Table 1. During the period, the amount of concentrate fodder and roughage fed each sheep was gradually increased and stabilized at 400 g and 1,000 g per day. At the end of this stage, seven sheep in each treatment group were randomly slaughtered and LD, BF and TB samples were collected. Meanwhile, the Mongolian sheep from each experimental group that were not slaughter began a 41-day minerals supplementation feeding phase and were renamed: the LCG group became the control group of the mineral supplementation feeding phase (SCG), the LCa group became the Ca supplementation group (SCa), the LZn group became the Zn supplementation group (SZn), the LCu group became the Cu supplementation group (SCu), the LCo group became the Co supplementation group (SCo), the LMn group became the Mn supplementation group (SMn), and the LSe group became the Se supplementation group (SSe). Each experimental group was supplemented with a mixed mineral salt as shown in Table 2 in their concentrate feed. Finally, all of the sheep were slaughtered to collect LD, BF and TB. A total of 98 samples of LD, 98 samples of BF and 98 samples of TB in this research.

**Table 1:** The components of mineral salts consumed in each experimental group during the mineral deficiency feeding phase.

**Items**	**LCG**	**LCa**	**LZn**	**LCu**	**LCo**	**LMn**	**LSe**
KCl, g/day	11.5	11.5	11.5	11.5	11.5	11.5	11.5
Na_2_SO_4_, g/day	9.0	9.0	9.0	9.0	9.0	9.0	9.0
NH_4_H_2_PO_4_, g/day	7.4	7.4	7.4	7.4	7.4	7.4	7.4
NaCl, g/day	5.0	5.0	5.0	5.0	5.0	5.0	5.0
MgO, g/day	1.7	1.7	1.7	1.7	1.7	1.7	1.7
FeSO_4_, mg/day	200.0	200.0	200.0	200.0	200.0	200.0	200.0
Ca(IO_3_)_2_, mg/day	1.4	1.4	1.4	1.4	1.4	1.4	1.4
CaCO_3_, g/day	10.0	0.0	10.0	10.0	10.0	10.0	10.0
ZnSO_4_, mg/day	132.0	132.0	0.0	132.0	132.0	132.0	132.0
MnSO_4_, mg/day	92.0	92.0	92.0	92.0	92.0	0.0	92.0
CuSO_4_, mg/day	36.0	36.0	36.0	0.0	36.0	36.0	36.0
CoSO_4_, mg/day	1.2	1.2	1.2	1.2	0.0	1.2	1.2
Na_2_SeO_3_, mg/day	1.0	1.0	1.0	1.0	1.0	1.0	0

LCG, control group of mineral deficiency feeding phase; LCa, Ca deficiency group; LZn, Zn deficiency group; LCu, Cu deficiency group; LCo, Co deficiency group; LMn, Mn deficiency group; LSe, Se deficiency group.

**Table 2:** The components of mineral salts consumed in each experimental group during the mineral supplementation feeding stage.

**Items**	**SCG**	**SCa**	**SZn**	**SCu**	**SCo**	**SMn**	**SSe**
KCl, g/day	11.5	11.5	11.5	11.5	11.5	11.5	11.5
Na_2_SO_4_, g/day	9.0	9.0	9.0	9.0	9.0	9.0	9.0
NH_4_H_2_PO_4_, g/day	7.4	7.4	7.4	7.4	7.4	7.4	7.4
NaCl, g/day	5.0	5.0	5.0	5.0	5.0	5.0	5.0
MgO, g/day	1.7	1.7	1.7	1.7	1.7	1.7	1.7
FeSO_4_, mg/day	200.0	200.0	200.0	200.0	200.0	200.0	200.0
Ca(IO_3_)_2_, mg/day	1.4	1.4	1.4	1.4	1.4	1.4	1.4
CaCO_3_, g/day	10.0	20.0	10.0	10.0	10.0	10.0	10.0
ZnSO_4_, mg/day	132.0	132.0	396.0	132.0	132.0	132.0	132.0
MnSO_4_, mg/day	92.0	92.0	92.0	92.0	92.0	276.0	92.0
CuSO_4_, mg/day	36.0	36.0	36.0	108.0	36.0	36.0	36.0
CoSO_4_, mg/day	1.2	1.2	1.2	1.2	3.6	1.2	1.2
Na_2_SeO_3_, mg/day	1.0	1.0	1.0	1.0	1.0	1.0	3.0

SCG, control group of minerals supplementation feeding phase; SCa, Ca supplementation group; SZn, Zn supplementation group; SCu, Cu supplementation group; SCo, Co supplementation group; SMn, Mn supplementation group; SSe, Se supplementation group.

### 2.2 Detection of the mineral composition in LD, BF, and TB

The fascia and fat were removed from LD, BF and TB, and subsequently homogenized using a mixer (PLODON HDD-807, USA). The homogenized samples were then stored in a plastic bag at −20°C until analysis.

The pretreatment of the sample was carried out according to the microwave digestion method specified in GB 5009.218-2016 (24). Briefly, 0.5 g of the muscle sample was mixed with 6 ml of nitric acid (65%, Sinopharm) and left in the polytetrafluoroethylene tube for 1 h. The samples were digested by microwave (REVO, LabTech, China) and the digestion parameters were listed in Table S2. After digestion, the samples were degassed by ultrasonication and fixed to 10 ml as the test solution.

According to our published methods (25), the contents of P, S, K, Ca, Mn, Fe, Co, Cu, Zn, and Se in the muscle samples were measured by the total reflection X-ray fluorescence spectrometry (S4·TSTAR, Burker, USA). Briefly, 200 μl of test solution was mixed with 20 μl of 0.2 g/L PVA (dispersant) and 2 μl of 1,000 μg/ml Ga standard solution (internal standard). With 10 μl absorbed, it was smeared evenly on the quartz slides and dried at 50°C. Then the test was performed and the test results were calibrated by the standard curve method (25).

### 2.3 Statistical analysis

All data were tested for normal distribution using SPSS 26.0 and then analyzed for significance by independent samples *t*-test (unpaired/ two-tailed), when *P*-value is <0.05 there is a significant difference.

## 3 Results

### 3.1 Minerals composition in muscles of Ca treatment

The content of P, S, K, Ca, Mn, Fe, Co, Cu, Zn, and Se in LD, BF, and TB was tested. The Co content in all treatment group samples was found to be below the detection limit of the instrument, and therefore, it was not displayed in the results.

Table 3 listed the minerals content in the LD, BF, and TB of the Ca treatment of Mongolian sheep. In LD, compared to the LCG group, the LCa group significantly increased the concentration of Ca but decreased the concentration of K (*P* < 0.05). Compared to the SCG group, the SCa group exhibited significant increases (*P* < 0.05) in the concentration of P and Cu. By contrast, the SCa group showed a substantial decrease (*P* < 0.05) in the concentration of Mn in LD.

**Table 3:** Minerals content in different muscles of Mongolian sheep of Ca treatment.

**Items**	**Longissimus dorsi**						**Biceps femoris**						**Triceps brachii**					
	**LCG**	**LCa**	***P*** **-value**	**SCG**	**SCa**	***P*** **-value**	**LCG**	**LCa**	***P*** **-value**	**SCG**	**SCa**	***P*** **-value**	**LCG**	**LCa**	***P*** **-value**	**SCG**	**SCa**	***P*** **-value**
P, mg/g	1.74 ± 0.05	1.67 ± 0.08	0.474	1.28 ± 0.04	1.47 ± 0.04	0.010	0.80 ± 0.08	0.63 ± 0.04	0.073	0.32 ± 0.06	0.89 ± 0.16	0.010	0.49 ± 0.07	0.62 ± 0.03	0.159	0.50 ± 0.10	0.51 ± 0.05	0.959
S, mg/g	1.68 ± 0.04	1.60 ± 0.09	0.29	1.39 ± 0.09	1.46 ± 0.03	0.466	0.84 ± 0.06	0.67 ± 0.05	0.047	0.37 ± 0.07	0.97 ± 0.17	0.007	0.58 ± 0.08	0.68 ± 0.02	0.196	0.68 ± 0.12	0.61 ± 0.06	0.600
K, mg/g	6.89 ± 0.19	5.75 ± 0.16	0.003	5.25 ± 0.26	5.70 ± 0.18	0.175	2.66 ± 0.15	0.28 ± 0.08	0.061	3.56 ± 0.50	2.86 ± 0.43	0.312	2.51 ± 0.19	2.52 ± 0.08	0.942	2.75 ± 0.27	1.85 ± 0.24	0.030
Ca, μg/g	14.5 ± 1.21	26.0 ± 1.18	0.011	11.7 ± 1.44	8.77 ± 0.62	0.109	5.01 ± 0.34	6.74 ± 0.50	0.016	5.99 ± 1.01	6.34 ± 0.78	0.789	8.33 ± 0.75	9.91 ± 0.64	0.135	10.2 ± 1.29	15.5 ± 1.21	0.011
Mn, μg/g	0.12 ± 0.02	0.08 ± 0.03	0.257	0.14 ± 0.02	0.07 ± 0.01	0.003	0.08 ± 0.01	0.19 ± 0.02	<0.001	0.09 ± 0.01	0.08 ± 0.01	0.840	0.09 ± 0.01	0.08 ± 0.01	0.469	0.08 ± 0.02	0.14 ± 0.01	0.021
Fe, μg/g	18.3 ± 0.92	17.1 ± 0.37	0.289	13.4 ± 0.57	12.6 ± 0.47	0.316	14.8 ± 0.84	16.5 ± 0.49	0.109	16.1 ± 0.78	15.5 ± 0.15	0.491	12.6 ± 0.57	14.8 ± 0.69	0.029	13.3 ± 0.34	17.9 ± 1.59	0.014
Cu, μg/g	1.03 ± 0.02	1.02 ± 0.06	0.814	0.68 ± 0.03	0.80 ± 0.03	0.016	0.47 ± 0.02	0.57 ± 0.02	0.003	0.45 ± 0.02	0.51 ± 0.02	0.124	0.43 ± 0.03	0.48 ± 0.02	0.118	0.42 ± 0.02	0.45 ± 0.03	0.420
Zn, μg/g	22.3 ± 0.44	21.0 ± 0.70	0.125	17.6 ± 1.23	19.5 ± 0.90	0.241	19.7 ± 0.51	19.4 ± 0.50	0.662	21.3 ± 1.04	24.0 ± 1.02	0.093	30.8 ± 1.14	30.8 ± 1.82	0.988	32.3 ± 1.15	33.6 ± 1.70	0.549
Se, μg/g	0.06 ± 0.006	0.06 ± 0.007	0.898	0.05 ± 0.004	0.06 ± 0.002	0.097	0.04 ± 0.001	0.03 ± 0.001	0.094	0.04 ± 0.003	0.04 ± 0.002	0.955	0.03 ± 0.002	0.04 ± 0.002	0.057	0.05 ± 0.003	0.05 ± 0.002	0.576

LCG, control group of mineral deficiency feeding phase; LCa, Ca deficiency group; SCG, control group of minerals supplementation feeding phase; SCa, Ca supplementation group.

In BF, compared to the LCG group, the LCa gruop significantly reduced the S content (*P* < 0.05) but increased the content of Ca, Mn, and Cu (*P* < 0.05). When compared with the SCG group, the SCa group only increased (*P* < 0.05) the content of P and S.

In TB, compared with the LCG group, the LCa group only significantly increased the content of Fe (*P* < 0.05). Compared to the SCG group, the SCa group had a noticeable impact on the content of four elements in sheep. The concentrations of Mn, Ca, and Fe significantly increased (*P* < 0.05). In addition, the level of K experienced a sharp decline (*P* < 0.05).

### 3.2 Minerals composition in muscles of Zn treatment

Table 4 displayed the changes in minerals content in the LD, BF, and TB of the Zn treatment of Mongolian sheep. Compared with the LCG group, LZn diet contributed to a significant increase (*P* < 0.05) in the content of Ca and Mn in LD. The content of S, K, Fe, and Zn in LD was less affected, showing a weak decline (*P* < 0.05). When compared with the SCG group, SZn group led to a significant increase (*P* < 0.05) only in the content of Cu and Zn for LD.

**Table 4:** Minerals content in different muscles of Mongolian sheep of Zn treatment.

**Items**	**Longissimus dorsi**						**Biceps femoris**						**Triceps brachii**					
	**LCG**	**LZn**	***P*** **-value**	**SCG**	**SZn**	***P*** **-value**	**LCG**	**LZn**	***P*** **-value**	**SCG**	**SZn**	***P*** **-value**	**LCG**	**LZn**	***P*** **-value**	**SCG**	**SZn**	***P*** **-value**
P, mg/g	1.74 ± 0.05	1.63 ± 0.03	0.082	1.28 ± 0.04	1.41 ± 0.06	0.101	0.80 ± 0.08	0.30 ± 0.05	<0.001	0.32 ± 0.06	1.22 ± 0.08	<0.001	0.49 ± 0.07	0.63 ± 0.07	0.205	0.50 ± 0.10	0.35 ± 0.07	0.275
S, mg/g	1.68 ± 0.04	1.56 ± 0.03	0.048	1.39 ± 0.09	1.45 ± 0.03	0.551	0.84 ± 0.06	0.38 ± 0.05	<0.001	0.37 ± 0.07	1.13 ± 0.13	<0.001	0.58 ± 0.08	0.64 ± 0.03	0.528	0.68 ± 0.12	0.48 ± 0.08	0.219
K, mg/g	6.89 ± 0.19	6.27 ± 0.12	0.017	5.25 ± 0.26	5.35 ± 0.12	0.722	2.66 ± 0.15	1.47 ± 0.22	<0.001	3.56 ± 0.50	3.06 ± 0.33	0.408	2.51 ± 0.19	2.71 ± 0.08	0.324	2.75 ± 0.27	1.97 ± 0.39	0.140
Ca, μg/g	14.5 ± 1.21	22.4 ± 1.09	<0.001	11.7 ± 1.44	9.13 ± 1.30	0.212	5.01 ± 0.34	10.1 ± 1.99	0.039	5.99 ± 1.01	17.3 ± 1.05	<0.001	8.33 ± 0.75	13.5 ± 0.56	<0.001	10.2 ± 1.29	15.2 ± 0.76	0.008
Mn, μg/g	0.12 ± 0.02	0.31 ± 0.06	0.009	0.14 ± 0.02	0.12 ± 0.02	0.395	0.08 ± 0.01	0.13 ± 0.01	0.016	0.09 ± 0.01	0.12 ± 0.02	0.227	0.09 ± 0.01	0.12 ± 0.01	0.066	0.08 ± 0.02	0.15 ± 0.03	0.088
Fe, μg/g	18.3 ± 0.92	15.6 ± 0.64	0.030	13.4 ± 0.57	14.8 ± 0.45	0.086	14.8 ± 0.84	15.5 ± 0.31	0.448	16.1 ± 0.78	16.5 ± 0.91	0.734	12.6 ± 0.57	13.3 ± 0.67	0.446	13.3 ± 0.34	17.2 ± 1.27	0.011
Cu, μg/g	1.03 ± 0.02	1.09 ± 0.06	0.427	0.68 ± 0.03	0.84 ± 0.02	0.001	0.47 ± 0.02	0.51 ± 0.03	0.252	0.45 ± 0.02	0.54 ± 0.02	0.017	0.43 ± 0.03	0.44 ± 0.03	0.729	0.42 ± 0.02	0.47 ± 0.02	0.105
Zn, μg/g	22.3 ± 0.44	20.4 ± 0.51	0.017	17.6 ± 1.23	21.8 ± 0.92	0.019	19.7 ± 0.51	20.6 ± 0.68	0.312	21.3 ± 1.04	24.0 ± 1.07	0.099	30.8 ± 1.14	30.8 ± 1.14	0.991	32.3 ± 1.15	34.4 ± 1.19	0.226
Se, μg/g	0.06 ± 0.006	0.05 ± 0.005	0.562	0.05 ± 0.004	0.06 ± 0.003	0.310	0.04 ± 0.001	0.03 ± 0.004	0.594	0.04 ± 0.003	0.05 ± 0.003	0.052	0.03 ± 0.002	0.04 ± 0.005	0.264	0.05 ± 0.003	0.05 ± 0.005	0.313

LCG, control group of mineral deficiency feeding phase; LZn, Zn deficiency group; SCG, control group of minerals supplementation feeding phase; SZn, Zn supplementation group.

In comparison with the LCG group, four macroelements of the LZn group were affected to a more significant extent. Specifically, the content of P, S, and K was significantly reduced (*P* < 0.05) for BF. In contrast, the content of Ca was significantly increased (*P* < 0.05). Among the trace elements, only Mn was significantly affected, showing an increase (*P* < 0.05). When compared with the SCG group, the content of 4 elements in BF was affected by SZn diet, showing a sharp rise, namely P, S, Ca, and Cu (*P* < 0.05).

In the LZn group, the concentration of Ca was increased significantly (*P* < 0.05) of TB compared with the LCG group. When compared with the SCG group, SZn group caused a sharp rise in the content of Ca and Fe (*P* < 0.05).

### 3.3 Minerals composition in muscles of Cu treatment

The changes in minerals content in the LD, BF, and TB of the Cu treatment of Mongolian sheep were documented in Table 5. The LCu diet had a significantly altered the minerals composition of LD. In comparison with the LCG group, the content of seven elements, namely P, S, K, Ca, Cu, Fe, and Zn were significantly reduced (*P* < 0.05). However, the content of Mn and Se remained unaffected by the LCu diet, showing no significant difference (*P* > 0.05). When compared to the SCG group, the SCu group caused a significant reduction (*P* < 0.05) only in the content of Mn and Ca for LD.

**Table 5:** Minerals content in different muscles of Mongolian sheep of Cu treatment.

**Items**	**Longissimus dorsi**						**Biceps femoris**						**Triceps brachii**					
	**LCG**	**LCu**	***P*** **-value**	**SCG**	**SCu**	***P*** **-value**	**LCG**	**LCu**	***P*** **-value**	**SCG**	**SCu**	***P*** **-value**	**LCG**	**LCu**	***P*** **-value**	**SCG**	**SCu**	***P*** **-value**
P, mg/g	1.74 ± 0.05	1.17 ± 0.02	<0.001	1.28 ± 0.04	1.42 ± 0.05	0.057	0.80 ± 0.08	0.77 ± 0.14	0.883	0.32 ± 0.06	0.29 ± 0.03	0.694	0.49 ± 0.07	0.27 ± 0.01	0.031	0.50 ± 0.10	0.47 ± 0.08	0.786
S, mg/g	1.68 ± 0.04	1.18 ± 0.03	<0.001	1.39 ± 0.09	1.37 ± 0.04	0.816	0.84 ± 0.06	0.81 ± 0.13	0.837	0.37 ± 0.07	0.37 ± 0.04	0.989	0.58 ± 0.08	0.39 ± 0.03	0.062	0.68 ± 0.12	0.76 ± 0.12	0.652
K, mg/g	6.89 ± 0.19	4.66 ± 0.13	<0.001	5.25 ± 0.26	5.21 ± 0.05	0.886	2.66 ± 0.15	2.56 ± 0.37	0.811	3.56 ± 0.50	1.20 ± 0.15	<0.001	2.51 ± 0.19	2.00 ± 0.36	0.256	2.75 ± 0.27	2.08 ± 0.39	0.198
Ca, μg/g	14.5 ± 1.21	7.61 ± 0.61	<0.001	11.7 ± 1.44	7.36 ± 1.01	0.037	5.01 ± 0.34	7.69 ± 0.47	<0.001	5.99 ± 1.01	4.21 ± 0.66	0.178	8.33 ± 0.75	8.49 ± 0.81	0.887	10.2 ± 1.29	9.41 ± 1.01	0.649
Mn, μg/g	0.12 ± 0.02	0.08 ± 0.02	0.145	0.14 ± 0.02	0.06 ± 0.02	0.013	0.08 ± 0.01	0.17 ± 0.03	0.004	0.09 ± 0.01	0.14 ± 0.02	0.055	0.09 ± 0.01	0.12 ± 0.01	0.021	0.08 ± 0.02	0.09 ± 0.01	0.681
Fe, μg/g	18.3 ± 0.92	12.2 ± 0.32	<0.001	13.4 ± 0.57	12.2 ± 0.20	0.095	14.8 ± 0.84	15.7 ± 0.54	0.371	16.1 ± 0.78	17.0 ± 0.58	0.379	12.6 ± 0.57	14.4 ± 0.18	0.012	13.3 ± 0.34	15.3 ± 0.50	0.005
Cu, μg/g	1.03 ± 0.02	0.63 ± 0.04	<0.001	0.68 ± 0.03	0.76 ± 0.04	0.137	0.47 ± 0.02	0.46 ± 0.01	0.652	0.45 ± 0.02	0.48 ± 0.01	0.381	0.43 ± 0.03	0.41 ± 0.03	0.686	0.42 ± 0.02	0.45 ± 0.02	0.312
Zn, μg/g	22.3 ± 0.44	15.8 ± 0.66	<0.001	17.6 ± 1.23	19.2 ± 0.73	0.286	19.7 ± 0.51	20.2 ± 0.62	0.549	21.3 ± 1.04	22.1 ± 1.03	0.596	30.8 ± 1.14	30.6 ± 1.43	0.907	32.3 ± 1.15	34.5 ± 1.83	0.321
Se, μg/g	0.06 ± 0.006	0.04 ± 0.002	0.084	0.05 ± 0.004	0.05 ± 0.003	0.565	0.04 ± 0.001	0.04 ± 0.003	0.865	0.04 ± 0.003	0.05 ± 0.004	0.162	0.03 ± 0.002	0.04 ± 0.003	0.018	0.05 ± 0.003	0.05 ± 0.002	0.926

LCG, control group of mineral deficiency feeding phase; LCu, Cu deficiency group; SCG, control group of minerals supplementation feeding phase; SCu, Cu supplementation group.

Compared with the LCG group, the Mn and Ca content of the LCu group was significantly increased (*P* < 0.05) in BF. When compared to the SCG group, only the content of K (*P* < 0.05) in BF was affected by SCu diet, showing a significant decrease.

In TB, the LCu group exhibited significant effects on 4 elements compared to the LCG group. To be specific, the concentration of P was reduced, while that of Mn, Se, and Fe were significantly increased (*P* < 0.05). When compared to the SCG group, only the concentration of Fe was significantly increased (*P* < 0.05) in the TB of the SCu group.

### 3.4 Minerals composition in muscles of Co treatment

The effect of Co treatment on the mineral composition of LD, BF, and TB is depicted in Table 6. Compared with the LCG group, the content of eight elements in LD was affected by the LCo diet, showing a sharp decline (*P* < 0.05), namely P, S, K, Ca, Mn, Fe, Cu, and Zn. Only the content of Se was unaffected by the LCo diet, with no significantly difference (*P* > 0.05). When compared with the SCG group, only the content of Mn in LD was affected by the SCo diet, showing a significant decrease (*P* < 0.05).

**Table 6:** Minerals content in different muscles of Mongolian sheep of Co treatment.

**Items**	**Longissimus dorsi**						**Biceps femoris**						**Triceps brachii**					
	**LCG**	**LCo**	***P*** **-value**	**SCG**	**SCo**	***P*** **-value**	**LCG**	**LCo**	***P*** **-value**	**SCG**	**SCo**	***P*** **-value**	**LCG**	**LCo**	***P*** **-value**	**SCG**	**SCo**	***P*** **-value**
P, mg/g	1.74 ± 0.05	1.33 ± 0.02	<0.001	1.28 ± 0.04	1.37 ± 0.03	0.08	0.80 ± 0.08	0.83 ± 0.08	0.775	0.32 ± 0.06	0.51 ± 0.06	0.046	0.49 ± 0.07	0.57 ± 0.02	0.407	0.50 ± 0.10	0.30 ± 0.03	0.087
S, mg/g	1.68 ± 0.04	1.24 ± 0.02	<0.001	1.39 ± 0.09	1.36 ± 0.04	0.767	0.84 ± 0.06	0.69 ± 0.05	0.107	0.37 ± 0.07	0.57 ± 0.06	0.043	0.58 ± 0.08	0.65 ± 0.03	0.448	0.68 ± 0.12	0.45 ± 0.05	0.110
K, mg/g	6.89 ± 0.19	5.08 ± 0.04	<0.001	5.25 ± 0.26	5.28 ± 0.14	0.922	2.66 ± 0.15	2.74 ± 0.28	0.818	3.56 ± 0.50	2.06 ± 0.08	0.009	2.51 ± 0.19	2.39 ± 0.15	0.648	2.75 ± 0.27	1.63 ± 0.23	0.009
Ca, μg/g	14.5 ± 1.21	6.60 ± 0.55	<0.001	11.7 ± 1.44	12.3 ± 1.42	0.770	5.01 ± 0.34	16.0 ± 1.51	<0.001	5.99 ± 1.01	14.9 ± 0.94	<0.001	8.33 ± 0.75	12.9 ± 0.55	<0.001	10.2 ± 1.29	12.4 ± 0.72	0.181
Mn, μg/g	0.12 ± 0.02	0.08 ± 0.01	0.013	0.14 ± 0.02	0.06 ± 0.02	0.005	0.08 ± 0.01	0.07 ± 0.01	0.316	0.09 ± 0.01	0.09 ± 0.01	0.743	0.09 ± 0.01	0.08 ± 0.01	0.195	0.08 ± 0.02	0.08 ± 0.01	0.843
Fe, μg/g	18.3 ± 0.92	12.6 ± 0.44	<0.001	13.4 ± 0.57	14.7 ± 0.67	0.183	14.8 ± 0.84	15.7 ± 0.71	0.440	16.1 ± 0.78	16.5 ± 0.46	0.624	12.6 ± 0.57	13.0 ± 0.38	0.591	13.3 ± 0.34	14.5 ± 0.76	0.169
Cu, μg/g	1.03 ± 0.02	0.80 ± 0.03	<0.001	0.68 ± 0.03	0.79 ± 0.06	0.126	0.47 ± 0.02	0.55 ± 0.02	0.005	0.45 ± 0.02	0.50 ± 0.03	0.213	0.43 ± 0.03	0.46 ± 0.02	0.261	0.42 ± 0.02	0.44 ± 0.02	0.564
Zn, μg/g	22.3 ± 0.44	18.1 ± 0.76	<0.001	17.6 ± 1.23	20.6 ± 0.80	0.066	19.7 ± 0.51	22.1 ± 1.06	0.067	21.3 ± 1.04	22.8 ± 1.02	0.350	30.8 ± 1.14	32.5 ± 1.27	0.346	32.3 ± 1.15	31.7 ± 1.67	0.788
Se, μg/g	0.06 ± 0.006	0.05 ± 0.005	0.250	0.05 ± 0.004	0.06 ± 0.003	0.259	0.04 ± 0.001	0.03 ± 0.002	0.220	0.04 ± 0.003	0.05 ± 0.002	0.143	0.03 ± 0.002	0.04 ± 0.002	0.073	0.05 ± 0.003	0.04 ± 0.003	0.504

LCG, control group of mineral deficiency feeding phase; LCo, Co deficiency group; SCG, control group of minerals supplementation feeding phase; SCo, Co supplementation group.

In BF, when compared with the LCG group, LCo treatment significantly increased (*P* < 0.05) the content of Ca and Cu. In comparison with the SCG group, a significant effect was exerted on 4 macroelements of the SCo group. To be specific, the content of P, S, Ca was significantly increased, while that of K was significantly reduced (*P* < 0.05).

Regarding TB, compared with the LCG group, the minerals was less affected for the LCo group. With only the concentration of Ca was significantly increased (*P* < 0.05), the other eight elements were unaffected by the LCo diet (*P* > 0.05). When compared with the SCG group, only the concentration of K was significantly reduced (*P* < 0.05) in the SCo.

### 3.5 Minerals composition in muscles of Mn treatment

Table 7 presented the impact of Mn treatment on the mineral composition of LD, BF, and TB in Mongolian sheep. LMn group contributed to a significant reduction (*P* < 0.05) in the content of K in LD compared with the LCG group. When compared with the SCG group, SMn group caused a sharp rise in the content of P and Cu for LD (*P* < 0.05).

**Table 7:** Minerals content in different muscles of Mongolian sheep of Mn treatment.

**Items**	**Longissimus dorsi**						**Biceps femoris**						**Triceps brachii**					
	**LCG**	**LMn**	***P*** **-value**	**SCG**	**SMn**	***P*** **-value**	**LCG**	**LMn**	***P*** **-value**	**SCG**	**SMn**	***P*** **-value**	**LCG**	**LMn**	***P*** **-value**	**SCG**	**SMn**	***P*** **-value**
P, mg/g	1.74 ± 0.05	1.65 ± 0.04	0.164	1.28 ± 0.04	1.46 ± 0.03	0.007	0.80 ± 0.08	0.55 ± 0.05	0.025	0.32 ± 0.06	0.28 ± 0.04	0.655	0.49 ± 0.07	0.78 ± 0.06	0.009	0.50 ± 0.10	0.32 ± 0.05	0.148
S, mg/g	1.68 ± 0.04	1.62 ± 0.04	0.380	1.39 ± 0.09	1.48 ± 0.07	0.453	0.84 ± 0.06	0.63 ± 0.06	0.029	0.37 ± 0.07	0.44 ± 0.07	0.446	0.58 ± 0.08	0.86 ± 0.06	0.015	0.68 ± 0.12	0.58 ± 0.12	0.555
K, mg/g	6.89 ± 0.19	6.38 ± 0.14	0.046	5.25 ± 0.26	5.80 ± 0.22	0.138	2.66 ± 0.15	2.43 ± 0.198	0.372	3.56 ± 0.50	1.70 ± 0.21	0.009	2.51 ± 0.19	3.01 ± 0.12	0.044	2.75 ± 0.27	1.71 ± 0.37	0.046
Ca, μg/g	14.5 ± 1.21	19.2 ± 1.99	0.076	11.7 ± 1.44	7.65 ± 1.19	0.059	5.01 ± 0.34	7.51 ± 1.09	0.066	5.99 ± 1.01	7.91 ± 1.00	0.213	8.33 ± 0.75	8.77 ± 1.08	0.750	10.2 ± 1.29	7.44 ± 1.57	0.201
Mn, μg/g	0.12 ± 0.02	0.23 ± 0.07	0.271	0.14 ± 0.02	0.11 ± 0.02	0.205	0.08 ± 0.01	0.09 ± 0.01	0.518	0.09 ± 0.01	0.09 ± 0.02	0.771	0.09 ± 0.01	0.05 ± 0.01	0.002	0.08 ± 0.02	0.08 ± 0.01	0.998
Fe, μg/g	18.3 ± 0.92	16.8 ± 0.49	0.183	13.4 ± 0.57	13.7 ± 0.30	0.619	14.8 ± 0.84	15.5 ± 0.55	0.516	16.1 ± 0.78	17.9 ± 1.79	0.333	12.6 ± 0.57	12.5 ± 0.25	0.917	13.3 ± 0.34	13.4 ± 0.30	0.766
Cu, μg/g	1.03 ± 0.02	0.99 ± 0.05	0.432	0.68 ± 0.03	0.88 ± 0.07	0.028	0.47 ± 0.02	0.57 ± 0.03	0.013	0.45 ± 0.02	0.50 ± 0.02	0.155	0.43 ± 0.03	0.46 ± 0.02	0.326	0.42 ± 0.02	0.46 ± 0.03	0.297
Zn, μg/g	22.3 ± 0.44	21.6 ± 1.32	0.606	17.6 ± 1.23	20.6 ± 0.93	0.090	19.7 ± 0.51	21.9 ± 0.71	0.030	21.3 ± 1.04	23.5 ± 0.74	0.132	30.8 ± 1.14	33.5 ± 1.82	0.230	32.3 ± 1.15	35.7 ± 0.97	0.049
Se, μg/g	0.06 ± 0.006	0.06 ± 0.005	0.615	0.05 ± 0.004	0.06 ± 0.003	0.385	0.04 ± 0.001	0.04 ± 0.003	0.659	0.04 ± 0.003	0.04 ± 0.002	0.725	0.03 ± 0.002	0.04 ± 0.003	0.066	0.05 ± 0.003	0.04 ± 0.003	0.250

LCG, control group of mineral deficiency feeding phase; LMn, Mn deficiency group; SCG, control group of minerals supplementation feeding phase; SMn, Mn supplementation group.

In BF of the LMn group, compared to the LCG group, the content of two macroelements were sharply reduced and that of two trace elements was significantly increased. Statistically, the content of P and S decreased, while that of Cu and Zn surged (*P* < 0.05). When compared with the SCG group, SMn group caused a steep decrease in only the content of K in BF (*P* < 0.05).

The LMn group exhibited significant changes in the concentration of four elements in TB compared with the LCG group. There was a significant increase in the concentration of three macroelements, including P, S, and K (*P* < 0.05). However, the concentration of Mn reduced significantly (*P* < 0.05). In comparison, the SMn group showed a sharp decrease in the concentration of K and an increase in Zn (*P* < 0.05) in TB. Notably, the concentration of K in TB of the LMn group showed the opposite trend to the SMn group.

### 3.6 Minerals composition in muscles of Se treatment

In Table 8, the impact of Se treatment on the mineral element composition of LD, BF, and TB in Mongolian sheep is presented. LSe diet significantly altered the minerals composition of LD. The content of seven elements, including P, S, K, Ca, Fe, Cu, and Se, was significantly reduced compared to the LCG group (*P* < 0.05). However, the content of Mn and Zn remained unaffected (*P* > 0.05) by the LSe diet. In comparison with the SCG group, SSe group decreased the content of Ca and increased the content of P and Cu in LD (*P* < 0.05).

**Table 8:** Minerals content in different muscles of Mongolian sheep of Se treatment.

**Items**	**Longissimus dorsi**						**Biceps femoris**						**Triceps brachii**					
	**LCG**	**LSe**	***P*** **-value**	**SCG**	**SSe**	***P*** **-value**	**LCG**	**LSe**	***P*** **-value**	**SCG**	**SSe**	***P*** **-value**	**LCG**	**LSe**	***P*** **-value**	**SCG**	**SSe**	***P*** **-value**
P, mg/g	1.74 ± 0.05	1.48 ± 0.01	0.010	1.28 ± 0.04	1.45 ± 0.03	0.004	0.80 ± 0.08	0.60 ± 0.09	0.135	0.32 ± 0.06	0.56 ± 0.08	0.036	0.49 ± 0.07	0.59 ± 0.06	0.364	0.50 ± 0.10	0.37 ± 0.10	0.356
S, mg/g	1.68 ± 0.04	1.43 ± 0.08	0.018	1.39 ± 0.09	1.48 ± 0.04	0.383	0.84 ± 0.06	0.65 ± 0.08	0.081	0.37 ± 0.07	0.63 ± 0.08	0.030	0.58 ± 0.08	0.70 ± 0.05	0.247	0.68 ± 0.12	0.48 ± 0.11	0.214
K, mg/g	6.89 ± 0.19	5.66 ± 0.28	0.003	5.25 ± 0.26	5.85 ± 0.09	0.417	2.66 ± 0.15	2.32 ± 0.14	0.131	3.56 ± 0.50	2.24 ± 0.20	0.028	2.51 ± 0.19	2.73 ± 0.14	0.376	2.75 ± 0.27	1.49 ± 0.23	0.006
Ca, μg/g	14.5 ± 1.21	6.91 ± 1.38	0.003	11.7 ± 1.44	5.55 ± 1.26	0.008	5.01 ± 0.34	8.03 ± 0.60	0.001	5.99 ± 1.01	5.72 ± 0.66	0.820	8.33 ± 0.75	14.2 ± 0.20	<0.001	10.2 ± 1.29	16.6 ± 1.30	0.007
Mn, μg/g	0.12 ± 0.02	0.08 ± 0.02	0.109	0.14 ± 0.02	0.17 ± 0.03	0.367	0.08 ± 0.01	0.09 ± 0.01	0.557	0.09 ± 0.01	0.24 ± 0.01	<0.001	0.09 ± 0.01	0.11 ± 0.01	0.181	0.08 ± 0.02	0.13 ± 0.02	0.130
Fe, μg/g	18.3 ± 0.92	12.6 ± 0.65	<0.001	13.4 ± 0.57	13.7 ± 0.76	0.763	14.8 ± 0.84	16.8 ± 1.33	0.225	16.1 ± 0.78	17.5 ± 0.38	0.134	12.6 ± 0.57	16.8 ± 2.34	0.087	13.3 ± 0.34	13.4 ± 0.33	0.764
Cu, μg/g	1.03 ± 0.02	0.81 ± 0.04	<0.001	0.68 ± 0.03	0.79 ± 0.02	0.014	0.47 ± 0.02	0.47 ± 0.02	0.983	0.45 ± 0.02	0.49 ± 0.02	0.308	0.43 ± 0.03	0.50 ± 0.08	0.340	0.42 ± 0.02	0.40 ± 0.01	0.250
Zn, μg/g	22.3 ± 0.44	19.3 ± 1.69	0.093	17.6 ± 1.23	17.8 ± 1.24	0.917	19.7 ± 0.51	21.3 ± 0.32	0.044	21.3 ± 1.04	22.9 ± 0.61	0.233	30.8 ± 1.14	34.0 ± 1.75	0.147	32.3 ± 1.15	32.9 ± 1.12	0.702
Se, μg/g	0.06 ± 0.006	0.03 ± 0.005	0.008	0.05 ± 0.004	0.06 ± 0.004	0.288	0.04 ± 0.001	0.0 ± 0.00	<0.001	0.04 ± 0.003	0.04 ± 0.002	0.227	0.03 ± 0.002	0.02 ± 0.0002	<0.001	0.05 ± 0.003	0.04 ± 0.004	0.202

LCG, control group of mineral deficiency feeding phase; LSe, Se deficiency group; SCG, control group of minerals supplementation feeding phase; SSe, Se supplementation group.

The feeding on Se deficiency diet had an immediate effect on Se content of sheep. The Se content in BF samples of the LSe group fell below the lower limit of detection, whereas it was 0.037 μg/g in BF of the LCG group. Among the other minerals, only Ca and Zn showed a significant increase in BF (*P* < 0.05). When compared with the SCG group, SSe group significantly reduced the content of K but increased the content of P, S, and Mn in BF (*P* < 0.05).

The LSe group exhibited a significant increase in the concentration of Ca and a reduction in Se concentration in TB (*P* < 0.05). In comparison with the SCG group, SSe group significantly increased the concentration of Ca and decreased that of K in TB (*P* < 0.05).

## 4 Discussion

The main objective of this study was to investigate the response of lamb meat to dietary minerals, as mutton is a significant source of minerals in human diets (1). Previous research has shown that meat-based diets tend to have lower Ca content (26), which was also confirmed in our study. Interestingly, our findings revealed that the Ca content in lamb meat does not appear to be regulated by the dietary Ca level. In our study, the Ca content in LD and BF was significantly higher in the Mongolian sheep of the LCa group compared to the LCG group with increases of 44.5 and 34.7%, respectively. Usually, animals can adapt to low Ca levels by reducing renal excretion of Ca, mobilizing Ca from the bone, and enhancing the gastrointestinal absorption of Ca (27). However, previous study has confirmed that the two mechanisms of reducing the renal excretion of Ca and enhancing the gastrointestinal absorption of Ca have not been observed in sheep (28). Therefore, the significant increase in muscle Ca content in the LCa group is likely attributed to the mobilization of Ca from bone, which is consistent with findings from other studies on animals adapting to low-Ca diets (29). Additionally, we also observed a significant increase of 14.6% in the content of P in LD after the Ca supplementation diet. This could be due to the dietary Ca:P ratio exceeding the recommendation ratio of 2:1 (30), resulting in an imbalance in the Ca:P ratio in Mongolian sheep. This imbalance may stimulate the organism to absorb more P and reduce P metabolism. Another possibility is the mobilization of P from bone precipitation (31), resulting in its accumulation in LD. It is important to note that long-term feeding of a diet with an imbalanced dietary Ca:P ratio can have adverse effects on the growth and mineral homeostasis of animals (30). This imbalance may also explain the decrease in Mn content in LD and K content in TB observed in our study.

In this study, the Zn concentration in LD was significantly lower in the LCG group and higher in the SCG group, after feeding on LZn and SZn diets, respectively. This indicates a positive correlation between the content of Zn in the diet and the Zn concentration in LD. Previous report has highlighted the role of Zn in muscle contraction and neuromuscular transmission, with skeletal muscle accumulating a significant amount of Zn (32). Therefore, a decrease in muscle Zn levels results from the inadequate Zn intake after the feeding on Zn-deficient diet. Conversely, the content of Zn in the muscle is significantly increased after the feeding on a Zn supplementation diet. Asli et al. (33) found a negative correlation between the concentration of Cu and that of Zn in camel muscle, our study revealed a significant increase of 23.7% in both Cu and Zn content in LD after the feeding on a Zn supplementation diet. This discrepancy may be related to different Cu requirements and the ability to accumulate Cu and Zn in different ruminant species (34). Furthermore, we observed that the content of Ca and Mn was significantly increased in LD and BF, and that of Ca in TB showing a significantly increased after feeding on a Zn deficiency diet. This could be due to the fact that Ca and Mn are primarily stored in bones and are more likely to be mobilized from bones to muscles when a Zn deficiency disrupts mineral balance in the body. Nejad et al. found that high levels of Ca in feed can inhibit Zn uptake (35), suggesting a possible competitive inhibitory relationship between Ca and Zn. The increase in Ca content in LD after the LZn treatment support this idea. The decrease in dietary Zn levels can impair various vital activities (36), and may have inhibited the accumulation of S, K, and Fe in the muscle. In addition, the content of Zn in BF and TB showed no difference from the LCG group or the SCG group, despite changes in dietary Zn level. This could be attributed to the differences in structure and function among different parts of the muscle, leading to variations in the capacity for mineral accumulation (22).

In this experiment, the content of Cu in LD was significantly decreased by 38.7% compared to the LCG group after feeding on the Cu deficiency diet, with no significant difference observed in the content of Cu in all muscles after feeding on the Cu supplementation diet. This may result from the impact caused by the unique digestive system of ruminants on the metabolism of Cu absorption. Cu deficiency diets meet the NRC recommendations on all minerals except for no supplementation of CuSO_4_. In particular, sulfur and molybdenum form thiomolybdate in the rumen, which can irreversibly bind to Cu and cannot be entertained by the intestine. When no exogenous Cu is added, thiomolybdate is absorbed through the rumen wall or small intestine into the bloodstream for combination with Cu in other compounds (34). This condition can exacerbate the symptoms of Cu deficiency in the ruminant, interferes with normal physiological activities in the body, and leads to a decreased ability to absorb and utilize other mineral nutrients. This mechanism may account for the notable decline in the concentrations of several minerals within the muscle subsequent to the consumption of diets lacking in copper. In addition, the high affinity of Cu bound to thiomolybdate may also explain why the muscle Cu content in sheep is unaffected by Cu supplementation diets. Excess Cu in the diet can affect the absorption and utilization of other minerals such as Ca, P, and Zn for animals (37). In this experiment, we also observed a significant reduction of 37.0% in Ca content after feeding on a Cu supplementation diet, which supports this argument. There was a research has been proposed that Cu and Mn share a common transporter protein (DMT 1) for intestinal absorption (38). Competition for this transporter protein could explain the significant increase in Mn content in BF and TB after feeding on a Cu deficiency diet.

We found that the Co content in all muscle samples fell below the limit of detection, indicating the difficulty for Co to accumulate in muscle, which is consistent with previous research (33). The synthesis of vitamin B12 by microorganisms in the rumen from Co in ruminants. When the concentration of Co in the rumen fluid falls below 0.5 mg/ml, the synthesis of vitamin B12 by the rumen flora is inhibited, which cause a series of related pathologies (39). The ruminants are more sensitive to vitamin B12 deficiency than non-ruminants, and it is more likely that the metabolic activities of carbohydrates, lipids, amino acids and DNA are affected (40). This may be the leading cause for the inhibited accumulation of P, S, K, Ca, Mn, Fe, Cu, and Zn in LD after the feeding on Co deficiency diet. This result also demonstrates the importance of Co as an essential trace element in the absorption and accumulation of other minerals. Ruminal microbial synthesis is the only natural source for ruminants to acquire vitamin B12. With 3% of the Co ingested by ruminants converted to VB12 by microorganisms, only 1% to 3% of the vitamin B12 produced is absorbed by the intestine into the bloodstream (41). The vitamin B12 is bound to transporter proteins in the bloodstream and transported to various organs and tissues such as the liver, kidneys and heart, for storage mainly in the liver (42). This may be the main reason why Co is difficult to detect in muscle even after the feeding on Co supplementation diet.

The concentration of Mn in the muscle did not show significant changes in sheep fed on both LMn and SMn diets. Similar to Ca, Mn is also a critical component of the bone structure that promotes the increase in bone mineral density (43), and the Mn present in the bone can also be mobilized to maintain Mn homeostasis in the body under the context of feeding on Mn deficiency diet. Pallauf et al. (44) found that the content of Mn in the muscle showed no increase after the Mn sulfate of a higher concentration was added to diets, which is consistent with the results of this study. Livestock have the ability to excrete excess Mn through bile secretion and feces, preventing the accumulation of high concentrations of Mn in their bodies, which could lead to poisoning (45). Interestingly, we discovered that the concentration of Zn in BF was significantly increased by 10.9% in the LMn group. This may be attributed to the improved absorptivity of Zn in the intestine after feeding on the Mn deficiency diet. Zinc and Mn are absorbed in the small intestine through the ZIP14 transporter protein. When the decrease in dietary Mn content reduces the flow of absorbable Mn through the intestine, it enhances the translocation and absorption of Zn by the ZIP14 protein (46), leading to the accumulation of Zn in the muscle.

For humans, meat products are the main source of dietary Se (47). The level of Se deposition in the muscle is considered an important indicator of Se levels in animals, and it is also suitable to indicate the adequacy of Se intake by humans (48). In this study, the content of Se in LD and TB of sheep fed on the Se deficiency diet was found significantly lower by 53.9 and 48.2%, respectively, than in LCG group, and not detected in BF. This is consistent with the study of Benes et al. (49) who indicated that the content of Se in the muscle decreased with the extension of feeding on Se deficiency diets. This may result from the fact that the Se in the muscle is preferentially transported to the blood to maintain the stability of Se in the blood when the animal is Se deficient (49). At the same time, the content of P, S, Ca, Fe, and Cu in LD significantly decreased by 14.6%, 14.4%, 52.3% 31.5%, and 21.4%, respectively, which may be that Se deficiency affected the normal development and function of muscle. Because Se deficiency in ruminants can lead to abnormalities in many physiological process, white myopathy and skeletal muscle degeneration and necrosis (50). In this study, the content of muscle of the three parts was found slightly higher than in the SCG group after feeding on the Se supplementation diet, despite no significant difference observed. These results are consistent with the results of published studies (51). This may be related to the absorption of Se by animals. Se enters the animal's body through the diet and initially accumulates in the intestine, with 30–100 days taken for other organ tissues to reach stable levels (52). In our experiment, the feeding on Se supplementation diet lasted roughly 41 days, during which the content of Se in the muscle may have remained elevated. Furthermore, we found that the content of Se in LD, BF and TB was only regulated by dietary Se and not affected by other elements, which confirmed the importance of Se supplementation in the diet. Ademi et al. (53) confirmed that Se supplementation had the strongest effect on the Se content in the blood of sheep. Dietary-based nutritional interventions aimed at increasing Se deposition in edible parts have been demonstrated as a reliable method to increase dietary Se intake in humans (51). Therefore, in order to produce Se-rich lamb meat, sufficient amount of Se must be supplemented in the diet.

In our study, the content of Fe in the LD was significantly reduced in the LCa, LZn, LCu, LCo, LMn, and LSe groups. On the other hand, the Fe content in the TB showed a significant increase in all groups. However, there was no significant change in the Fe content in the BF across all groups. There was a study has been proposed that the absorption of Fe is largely dependent on the animal's body needs (54). LD, BF, and TB may have different requirements and accumulation capacity for Fe, which may be related to the different functions that the different muscles have to perform. Each muscle has specific roles and metabolic demands, which may influence their Fe uptake and utilization. Further research is needed to better understand the mechanisms underlying these differences in Fe content among different muscle groups.

## 5 Conclusion

The response of the concentration of various minerals in Mongolian sheep muscles to the mineral content of the diet is a complex process, but we can still observe some obvious patterns. The content of Se in LD, BF, and TB being only affected by Se in the diet. The Zn content in LD is closely correlated with the dietary Zn levels, and the multiple minerals content varies with the changes in dietary Cu levels. Additionally, the Ca content in grazing lamb meat does not appear to follow a clear pattern with the level of Ca in the diet, which may require further research and exploration. As a whole, there is a close relationship between the mineral content in the diet and the mineral nutrient composition of grazing lamb meat. To achieve the production of high-quality grazing lamb meat that is rich in mineral nutrients, it is crucial to have a comprehensive understanding of these interconnections.

## Data availability statement

The original contributions presented in the study are included in the article/Supplementary material, further inquiries can be directed to the corresponding author.

## Ethics statement

All the animal-related procedures involved in this study were approved by the Animal Care and Use Committee of Inner Mongolia University (IMU-sheep-2020-040). The study was conducted in accordance with the local legislation and institutional requirements.

## Author contributions

LMe: Writing—original draft. XJ: Writing—original draft. ZQ: Data curation, Visualization, Writing—review & editing. LMi: Writing—review & editing.
