# Supplementary material for: Effects of dietary minerals deficiency and supplementation on different parts of muscle minerals content in grazing Mongolian sheep
Source: Front Vet Sci. 2024 Jan 22;11:1301852. doi: 10.3389/fvets.2024.1301852 (PMC10845341; doi:10.3389/fvets.2024.1301852)
Supplement: Supplementary file 1 [file Table_1.docx]

Supplementary Material

# Supplementary Figures and Tables

| **Items** | **Oats** | **Mixed forage** |
| --- | --- | --- |
| Macro-elements, mg/g | | |
| P | 2.40 | 0.65 |
| S | 1.39 | 1.09 |
| K | 5.05 | 11.83 |
| Ca | 0.52 | 7.43 |
| Micro-elements, μg/g | | |
| Mn | 41.20 | 53.57 |
| Fe | 112.15 | 566.05 |
| Cu | 3.49 | 4.48 |
| Co | -^1^ | - |
| Zn | 21.34 | 16.77 |
| Se | 0.09 | - |

**Table S1** The composition of nutrients in oats and mixed forage

^1^-: Below the detection threshold.

**Table S2** The program of the graphite digester

| **Step** | **Temperature** | **Rising time** | **Holding time** |
| --- | --- | --- | --- |
| 1 | 120℃ | 5 minutes | 5 minutes |
| 2 | 150℃ | 5 minutes | 5 minutes |
| 3 | 190℃ | 5 minutes | 20 minutes |
